# The influence of BCG vaccine strain on mycobacteria-specific and non-specific immune responses in a prospective cohort of infants in Uganda

**DOI:** 10.1016/j.vaccine.2012.01.053

**Published:** 2012-03-09

**Authors:** Elizabeth J. Anderson, Emily L. Webb, Patrice A. Mawa, Moses Kizza, Nancy Lyadda, Margaret Nampijja, Alison M. Elliott

**Affiliations:** a Imperial College London School of Public Health, London W2 1PG, UK; b London School of Hygiene & Tropical Medicine, London WC1E 7HT, UK; c MRC/UVRI Uganda Research Unit on AIDS, P.O. Box 49, Entebbe, Uganda; d Entebbe Hospital, P.O. Box 29, Entebbe, Uganda

**Keywords:** BCG, Strain, Immune response, Non-specific effects, BCG scar

## Abstract

**Background:**

Globally, BCG vaccination varies in efficacy and has some non-specific protective effects. Previous studies comparing BCG strains have been small-scale, with few or no immunological outcomes and have compared TB-specific responses only. We aimed to evaluate both specific and non-specific immune responses to different strains of BCG within a large infant cohort and to evaluate further the relationship between BCG strain, scarring and cytokine responses.

**Methods:**

Infants from the Entebbe Mother and Baby Study (ISRCTN32849447) who received BCG-Russia, BCG-Bulgaria or BCG-Denmark at birth, were analysed by BCG strain group. At one year, interferon-gamma (IFN-γ), interleukin (IL)-5, IL-13 and IL-10 responses to mycobacteria-specific antigens (crude culture filtrate proteins and antigen 85) and non-mycobacterial stimuli (tetanus toxoid and phytohaemagglutinin) were measured using ELISA. Cytokine responses, scar frequency, BCG associated adverse event frequency and mortality rates were compared across groups, with adjustments for potential confounders.

**Results:**

Both specific and non-specific IFN-γ, IL-13 and IL-10 responses in 1341 infants differed between BCG strain groups including in response to stimulation with tetanus toxoid. BCG-Denmark immunised infants showed the highest cytokine responses. The proportion of infants who scarred differed significantly, with BCG scars occurring in 52.2%, 64.1% and 92.6% of infants immunised with BCG Russia, BCG-Bulgaria and BCG-Denmark, respectively (*p* < 0.001). Scarred infants had higher IFN-γ and IL-13 responses to mycobacterial antigens only than infants without a scar. The BCG-Denmark group had the highest frequency of adverse events (*p* = 0.025). Mortality differences were not significant.

**Conclusions:**

Both specific and non-specific immune responses to the BCG vaccine differ by strain. Scarring after BCG vaccination is also strain-dependent and is associated with higher IFN-γ and IL-13 responses to mycobacterial antigens. The choice of BCG strain may be an important factor and should be evaluated when testing novel vaccine strategies that employ BCG in prime–boost sequences, or as a vector for other vaccine antigens.

## Highlights

► Largest study comparing BCG strains and first to assess strain effects on non-specific responses. ► Cytokine responses to both mycobacterial and non-mycobacterial stimuli are strain-dependent. ► BCG-Denmark causes higher cytokine levels and more scars and adverse events than two other strains. ► Sex may interact with the effect of strain; non-specific responses are not associated with scars. ► BCG strain choice may be important and should be evaluated in novel vaccine strategies using BCG.

## 1 Background

BCG (Bacille Calmette–Guérin), derived from *Mycobacterium bovis* in 1926 [1], is the most widely administered vaccine in the world, with 90.8% global coverage in 2009 [2]. Several phenotypically diverse strains are in use, arising from independent subculture of attenuated mycobacteria in laboratories across the world [3–5]. Reported efficacy of BCG has varied considerably, ranging from 0 to 80% [6–8], with tropical countries reporting lower protection against tuberculosis [8,9]. Several factors that vary with latitude may alter BCG potency, including exposure to environmental mycobacteria [6] and other common infections in the tropics [10]. Although BCG strain alone cannot account for the extent of variation in efficacy [8], it may account for some of the variation observed in common clinical and immunological outcomes used in research, such as BCG scarring and cytokine responses.

The ongoing problem of establishing an accurate immunological proxy for protection against TB complicates BCG efficacy studies. Whilst the production of IFN-γ by CD4+ T cells is vital [20,21], it is not sufficient, and a more complex picture is emerging involving multiple cytokines and T cell subsets, including regulatory T cells [20–24]. Several small human studies have compared immune responses to different BCG strains, with variable results [11–19], including two in Africa, which demonstrated some variability in T cell proliferation and interferon-gamma (IFN-γ) production depending on the strain and route of administration [11–13].

Besides TB, there is evidence that BCG may also provide protection against other illnesses, with studies showing lower rates of malaria, acute lower respiratory tract infection and overall mortality in BCG-immunised individuals [14,25–27]. Such non-specific effects of BCG have also been demonstrated immunologically, with increased cytokine responses to both BCG-related antigens and non-BCG antigens such as tetanus toxoid (TT) or phytohaemagglutinin (PHA) amongst BCG-immunised children [28,29].

Our large birth cohort in Entebbe, Uganda, provided an opportunity to examine whether different BCG strains elicited different immune responses to both mycobacterial and non-mycobacterial stimuli, and to evaluate further the relationship between BCG strain, scarring and cytokine responses.

## 2 Methods

### 2.1 Study design

The Entebbe Mother and Baby Study, a randomised double-blind, placebo-controlled trial of the effect of anthelminthic treatment on responses to childhood immunisations, has been described elsewhere [10,30–32]; the following methods are relevant to this analysis.

### 2.2 Recruitment and follow-up

Pregnant women from the Entebbe peninsula in Uganda were screened and enrolled at Entebbe Hospital from April 2003 until November 2005. Socio-demographic details were obtained by questionnaire during antenatal care. Stool and blood samples were taken for parasitological and HIV testing and babies of participating mothers were followed up. Second-born twins, babies who had not received all three doses of tetanus vaccine and babies who had received their BCG after 6 months or outside Entebbe Hospital (where strain data were unavailable) were excluded from this analysis. The HIV status of HIV-exposed infants was ascertained through PCR of blood taken at age 6 weeks and rapid-test serology performed at 18 months.

At age 12 months infants had blood taken for immunological analysis; anthropometric parameters and the presence and diameter of BCG scars were documented. If unwell at the time of the visit, infants were treated accordingly and the study investigations were conducted up to 2 months later. Throughout the study the clinic was freely accessible as required, with any illnesses or vaccine-related adverse events being recorded.

### 2.3 Immunisation

Vaccines were provided by Uganda National Medical Stores. Nurses at Entebbe Hospital immunised babies at birth with 0.05 ml of intradermal BCG over the right deltoid, containing the strain and lot available at the time.^2^ The three strains used during the study period were BCG-Russia (BCG-I strain from Moscow, Serum Institute of India, India); BCG-Bulgaria (BCG-SL 222 Sofia strain, BB-NCIPD Ltd., Bulgaria); and BCG-Denmark (BCG-SSI 1331, Statens Seruminstitut, Denmark). Other vaccines administered were OPV (at 0, 6, 10 and 14 weeks); DPT, Hib and Hep B (at 6, 10 and 14 weeks); and measles (at 9 months).

### 2.4 Immunological analysis

Cytokine responses were assessed by six-day whole blood culture and ELISA assay, as previously described [10]. Cytokine levels in culture supernatants were measured by ELISA (Beckton Dickinson, UK) after stimulation by crude culture filtrate protein, antigen 85 (cCFP, Ag 85; Colorado State University, USA), tetanus toxoid (TT; Statens Seruminstitut, Denmark) and phytohaemagglutinin (PHA; Sigma, UK). CFP and Ag85 were used to assess mycobacteria-specific immune responses and PHA and TT to assess non-specific effects of BCG strains.

IFN-γ and IL-10 were analysed as representative of type 1 and regulatory activity respectively. Although IL-4 levels are central to the type 2 response, IL-5 and IL-13 are more detectable in supernatants and were therefore measured instead. Results were adjusted according to responses in unstimulated wells. To avoid time dependent effects of assay performance, the sequentially collected samples were tested in a randomised order.

### 2.5 Statistical analysis

Statistical analyses were conducted using Stata/IC 11.1. Infants were grouped according to strain of BCG received. Characteristics of the three groups of infants and mothers were compared using Pearson's chi-squared test for categorical variables and the *t*-test for continuous variables. Cytokine levels below the threshold of detection were set to zero^3^; distributions of cytokine results were highly skewed, a recognised phenomenon in immunological studies [10,30,33]. Cytokine results were therefore transformed to log_10_(concentration + 1) before analysis. Mean cytokine responses were compared between strain groups using random effects linear regression, anti-logging the regression coefficients to obtain geometric mean ratios (GMRs). Random effects were used to account for potential between-lot variability (since several lots of vaccine were administered within each BCG strain group). As some cytokine results remained skewed after log_10_ transformation, analyses were boostrapped [33] with 10,000 repeats to calculate bias-corrected accelerated confidence intervals. Cytokine responses of infants with and without a BCG scar were compared using the same methods but without random effects (being independent of potential between-lot variability).

Odds ratios for associations between BCG strain and scar presence were calculated through random effects logistic regression. BCG scar sizes were compared across strain groups through linear regression. Because differences in male and female infant responses to BCG have been reported [10,14,26,34,35] all analyses were repeated stratifying by sex, using Wald tests to assess interactions between the effects of sex and strain.

Consistency of results was checked between different batches of assay antigen. The second batches of cCFP and TT appeared to produce slightly different cytokine responses. The second batch of cCFP was only used in a small number of samples, which were therefore excluded from analysis. However, the groups tested with different batches of TT were of similar size and therefore cytokine responses to TT were adjusted for TT batch to avoid loss of power.

As different strains were administered during set periods of time in sequence according to their availability, there was potential for confounding by factors associated with both calendar time and cytokine responses (Table 1) [10]. Factors considered as *a priori* confounders were infant malaria parasitaemia, maternal *Mansonella perstans* and hookworm infection, and area of residence (as the recruitment area was gradually expanded to include more rural areas surrounding Entebbe and different environmental exposures may influence cytokine responses). All analyses were adjusted for the above factors as well as HIV infection, which causes severe restriction of infant cytokine responses [10,36]. As anthelminthic treatment allocation was randomised and was found to have no effect on infant immune responses [30], maternal anthelminthic was not considered as a possible confounder, or adjusted for, in this analysis.

Mortality rates per 1000 person years were compared between strain groups using Cox regression hazard ratios. The numbers of BCG-related adverse events were tabulated by group and compared using Fisher's exact test.

### 2.6 Ethics statement

All mothers gave informed, written consent. Ethical approval for the trial was granted from the Science and Ethics Committee of the Uganda Virus Research Institute, Uganda National Council for Science and Technology, and London School of Hygiene and Tropical Medicine.

## 3 Results

### 3.1 Participants

Of 2345 livebirths, 2081 singleton babies received BCG at Entebbe Hospital within 6 months of birth. Of these, 145 infants did not have data on immunisations other than those administered at birth; 220 infants did not receive all three doses of tetanus toxoid; 60 infants died or were lost to follow-up before 1 year of age; 315 infants were still in follow-up but did not provide a blood sample within the specified time frame. Therefore 1341 samples with immunological results were eligible for this analysis. Mothers of infants not included were in earlier stages of gestation at recruitment, younger, and more likely to be first-time mothers, of lower socio-economic status and living in a more distant study area [30]. However, lack of eligibility was not associated with strain group.

The three groups had similar socio-demographic characteristics (Table 1); there were however differences in maternal hookworm and *M. perstans* infection prevalence. Amongst infants, there were no significant differences in sex ratio, birth weights, HIV status or mean lymphocyte counts; but malaria parasitaemia differed between groups.

### 3.2 Cytokine responses and scar frequency

Cytokine responses to both mycobacteria-specific (cCFP and Ag85) and non-specific stimuli (TT and PHA) differed between BCG strains (Table 2). In particular, the BCG-Denmark group demonstrated IFN-γ responses that were significantly higher than those of the BCG-Russia group to all four stimuli, as well as higher IL-13 responses to cCFP and PHA. Compared to BCG-Russia, IL-5 responses did not differ in the BCG-Denmark group. However in the BCG-Bulgaria group, they were marginally lower in response to specific antigens. IL-10 levels were notably higher for both BCG-Bulgaria and BCG-Denmark groups relative to BCG-Russia in response to all stimuli.

Overall, 59.0% of the one-year olds had a BCG scar. There were significant differences between the proportions of each group who had a BCG scar: BCG-Denmark had a markedly higher association with scarring than BCG-Russia or BCG-Bulgaria (*p* < 0.001; Table 2). BCG scar size did not significantly differ between groups (data not shown).

The above observations were similar after stratifying by infant sex. For cCFP, Ag85 and PHA there was a tendency for some effects of BCG strain to appear stronger in female infants (data not shown). In response to TT, there was an interaction between sex and strain for IL-10 responses (Table 3), with stronger associations amongst female infants. However, similar proportions of girls and boys developed a scar.

Samples from infants with BCG scars demonstrated higher IFN-γ and IL-13 responses to mycobacterial antigens, but not to TT or PHA, than those without a scar (Table 4). There were no differences in IL-5 or IL-10 responses by scar status for any stimulus.

### 3.3 BCG-related adverse events and mortality from all causes

BCG-related adverse events included 2 ulcers and 12 abscesses, occurring in 0.3% of the BCG-Russia group, 1.0% of the BCG-Bulgaria group and 1.8% of the BCG-Denmark group (*p* = 0.025). Observed mortality appeared slightly higher in the BCG-Denmark group, however the study was underpowered to detect significant differences (Table 5).

## 4 Discussion

This infant cohort in a low-resource tropical country, recruited before birth and followed up prospectively, provided a good opportunity to investigate potential differences between the effects of three BCG strains that are commonly used globally. We found significant differences in mycobacteria-specific and non-specific immune responses, and in the frequency of BCG-associated adverse events, according to the vaccine strain used. To our knowledge, this is the largest study to evaluate the effects of BCG strain on immune responses to the BCG vaccine and the only study to assess both specific and non-specific responses [11].

Other studies have shown that BCG elicits type 1 and type 2 responses, to both mycobacteria-specific and non-specific stimuli [28,29]. Non-specific effects have also been reported at the clinical level, with reductions in non-TB infections and in overall mortality in BCG immunised infants [14,25–27,35,36]. Furthermore, BCG has been shown to act non-specifically as a primer for other vaccines [29]. Here we were able to conduct a broad analysis of the effect of BCG strain by comparing type 1 (IFN-γ), type 2 (IL-5 and IL-13) and regulatory (IL-10) responses to both mycobacteria-specific (cCFP and Ag85) and non-specific (TT and PHA) stimuli.

The results revealed three significant patterns of strain-dependent variability of immune responses to both mycobacteria-specific and non-specific stimuli: higher IFN-γ and IL-13 responses in the BCG-Denmark group; lower IL-5 responses in the BCG-Bulgaria group; and higher IL-10 responses in both the BCG-Denmark and BCG-Bulgaria group compared to BCG-Russia. Consistent with being at the greatest genetic distance from the other two strains [9], the cytokine responses of the BCG-Denmark group were the most divergent. Surprisingly however, they were also the highest overall, despite being most distantly related to the original *M. bovis* strain [37]. It is also interesting that BCG-Bulgaria and BCG-Russia behaved slightly differently in this cohort, despite being genetically identical, except for possible single nucleotide changes [38]. As all infants were immunised with BCG, it is uncertain how these findings would relate to non-specific responses (such as the response to TT) amongst BCG-unvaccinated infants, however, differences between strains in non-specific effects were clearly demonstrated.

It is possible that the greater immunogenicity of BCG-Denmark may lead to better protection against TB. However, IFN-γ alone is an insufficient protective marker and it is feasible that higher regulatory IL-10 production in the same group may counteract its effects [39]. The observation that IL-10 production differed between strains is contrary to a recent study [28] that found that BCG did not stimulate an IL-10 response. This analysis suggests that the ability of BCG to stimulate an IL-10 response may be strain-dependent, although a study that compared BCG-Denmark to BCG-Brazil and BCG-Japan, found no such differences [16]. Importantly, the differences across groups were observed in response to TT and PHA as well as to mycobacterial antigens, suggesting that the non-specific effects of BCG immunisation are likely to be dependent on the strain administered. The finding for TT specifically indicates that BCG strain differences can modulate the infant response to subsequent, unrelated exposures to antigens, including vaccines (and presumably, pathogens).

There was striking disparity in BCG scar frequency between groups, with an almost two-fold increase in scarring frequency in the BCG-Denmark group compared to the BCG-Russia group. The overall proportion with scars was 59%, despite 100% immunisation coverage at birth. This is much lower than observed scar prevalence in other populations [25,27,28,40] and is unlikely to be attributable to poor immunisation technique considering that 93% of the BCG-Denmark group developed a scar (*p* < 0.001). This analysis may be evidence that the association between BCG scar frequency and immunisation status is strain-dependent. BCG scars have often been used in research to identify BCG immunised individuals, which may be a valid method in a population uniformly immunised with one strain, such as BCG-Denmark, which causes the majority of vaccinees to scar. However, in populations immunised with a strain that causes fewer scars, scarring may reflect an individual's immune response to the vaccine rather than immunisation status, leading to many misclassifications. In countries using multiple strains, identifying individuals by scar status may give results reflecting the effects of one strain and not the whole immunised population. Although correlations between scar size and cytokine responses have been demonstrated at 4 years of age [28], it is unsurprising that no relationship was shown here, as BCG scars are still very small at one year.

Studies in Guinea Bissau have demonstrated an association between scar development after BCG immunisation and benefiting from its non-specific effects [14,25–27]. However, our results show no correlation between scarring and non-specific cytokine responses, with only higher mycobacteria-specific IFN-γ and IL-13 responses differentiating those with a scar from those without. BCG strain did influence both non-specific immune responses and scar development, suggesting that BCG strain could be a confounder in the relationship between scarring and non-specific responses. For example, the BCG-Denmark strain caused both higher IFN-γ responses to non-specific stimuli and also a greater frequency of scarring. The infants’ sex modified the effect of BCG strain on responses to tetanus toxoid, but not to either mycobacteria-specific antigen. This finding is in keeping with reports that girls may experience more non-specific BCG effects than boys [14,26,35,36] although a mechanism for this phenomenon has not been established [36].

This study was underpowered to detect differences in mortality. However, significant differences were detected between the proportions of each group that experienced an adverse event, the highest of which occurred in the BCG-Denmark group. As BCG-Denmark stimulated the highest cytokine responses, it is possible that there may be a trade-off between immunogenicity and adverse event induction, although the small number of events warrants caution in interpreting this relationship.

Our results emphasise the importance of identifying and adjusting for the strain of BCG used in studies of vaccine efficacy, or of correlates of protection, whenever BCG is employed as part of a vaccination strategy. This includes studies evaluating novel vaccines that employ a prime–boost strategy, as the choice of priming BCG strain may influence the results. Our data also suggests that BCG strain may influence outcomes when employing BCG as a vector for vaccines against other pathogens. Importantly, the choice of BCG strain may have clinical effects beyond the protection against TB. Further large-scale comparative investigation of BCG strains with clinical primary outcomes would be valuable.

This analysis was not part of our original trial design, so infants were not randomised to receive different BCG strains. This may have led to potential confounders, for example, due to different seasonal exposures to infections, which we could not account for. However, we did identify differences in maternal helminth and infant malaria status between the groups and we adjusted for these variables in the analysis; adjusted results were similar to crude findings. One-year olds were appropriate subjects as it has been shown that IFN-γ, IL-5, IL-13 and IL-10 responses to BCG given at birth are detectable at one year with some effects waning by two years [28]. However, it was not possible to analyse TB outcomes or long-term effects. Further work will include a repeated analysis of the same cohort at five years, assessing TB prevalence and incidence as well as non-TB illnesses and overall mortality. This may provide the warranted longitudinal evidence of whether or not strain-dependent effects observed at the molecular level translate to clinical outcomes in this cohort. In the meantime, whenever multiple BCG strains are used in future research, or when the effects of BCG or other immunisation regimes are compared in different populations, accounting for BCG strain is vital.

## Figures and Tables

**Table 1:** Maternal and infant characteristics according to BCG-strain groups.

	BCG-Russia (*n* = 1124)	BCG-Bulgaria (*n* = 788)	BCG-Denmark (*n* = 169)	*p*-Value^b^
	Frequency (%)^a^	Frequency (%)^a^	Frequency (%)^a^	
**Maternal characteristics**				
Age (years)				
<20	279 (25%)	183 (23%)	44 (26%)	0.765
20–24	431 (38%)	298 (38%)	55 (33%)	
25–29	248 (22%)	184 (23%)	41 (24%)	
30–34	108 (10%)	89 (11%)	20 (12%)	
≥35	58 (5%)	34 (4%)	9 (5%)	
Education (4 missing values)				
None	35 (3%)	35 (4%)	5 (3%)	0.748
Primary	579 (52%)	398 (51%)	83 (49%)	
Secondary	418 (37%)	287 (37%)	68 (40%)	
Tertiary	92 (8%)	65 (8%)	12 (7%)	
Tribe (1 missing value)				
Muganda	564 (50%)	388 (49%)	84 (50%)	0.596
Munyankole	98 (9%)	77 (10%)	15 (9%)	
Mutoro	43 (4%)	36 (5%)	7 (4%)	
Musoga	48 (4%)	27 (3%)	9 (5%)	
Luo	71 (6%)	38 (5%)	7 (4%)	
Munyawanda	60 (5%)	53 (7%)	5 (3%)	
Others	240 (21%)	168 (21%)	42 (25%)	
Study area (28 missing values)				
Entebbe	470 (42%)	324 (42%)	62 (37%)	0.093
Kigungu	131 (12%)	83 (11%)	19 (11%)	
Manyago, Kibale	330 (30%)	205 (27%)	46 (27%)	
Katabi, roadside	97 (9%)	92 (12%)	23 (14%)	
Katabi, rural	85 (8%)	68 (9%)	18 (11%)	
Household socioeconomic status (40 missing values)				
1 (low)	71 (6%)	34 (4%)	10 (6%)	0.323
2	104 (9%)	67 (9%)	13 (8%)	
3	347 (31%)	248 (32%)	45 (27%)	
4	296 (27%)	241 (31%)	48 (29%)	
5	227 (21%)	140 (18%)	41 (25%)	
6 (high)	59 (5%)	41 (5%)	9 (5%)	
Gravidity				
1	303 (27%)	209 (27%)	46 (27%)	0.933
2–4	639 (57%)	440 (56%)	93 (55%)	
≥5	182 (16%)	139 (18%)	30 (18%)	
Malaria parasitaemia (37 missing values)				
Positive	102 (9%)	89 (11%)	22 (13%)	0.197
HIV status				
Positive	130 (12%)	94 (12%)	17 (10%)	0.788
Helminth infections (6 missing values)				
Hookworm	534 (48%)	302 (38%)	70 (41%)	**<0.001**
*Schistosoma mansoni*	229 (20%)	136 (17%)	26 (15%)	0.105
*Mansonella perstans*	232 (21%)	160 (20%)	50 (30%)	**0.023**

**Infant characteristics**				
Sex				
Male	566 (50%)	412 (52%)	90 (53%)	0.617
Age at BCG immunisation (days)				
<1	257 (23%)	197 (25%)	38 (22%)	0.274
1–2	647 (58%)	417 (53%)	99 (59%)	
3–7	79 (7%)	75 (10%)	11 (7%)	
8–28	81 (7%)	57 (7%)	16 (9%)	
29–6 months	60 (5%)	42 (5%)	5 (3%)	
Malaria parasitaemia at 1 year (655 missing values)^c^				
Positive	53 (7%)	18 (3%)	9 (7%)	**0.033**
HIV status at 1 year (43 missing values)^c^^,^^d^				
Positive	24 (2%)	21 (3%)	6 (4%)	0.478

	Mean (±SD)	Mean (±SD)	Mean (±SD)	
Birth weight (326 missing values) (kg)	3.186 (±0.476)	3.171 (±0.495)	3.179 (±0.462)	0.827
Lymphocyte count at 1 year (544 missing values)^c^ (cells per 10^9^/l)	6.927 (±2.683)	6.870 (±2.607)	6.625 (±2.414)	0.474

*p*-Values <0.05 are indicated in bold.

a indicates crude frequencies and proportions (%) of each variable outcome within each BCG strain group, unless otherwise indicated.

b *p*-Value for test of null hypothesis that there is no association between characteristic and BCG strain group.

c Data are only available for infants who attended the study clinic at 1 year.

d Babies were assumed to be HIV-negative if their mothers were HIV-negative, and to be HIV positive at one year if serological results were positive at age 18 months.

**Table 2:** Effect of BCG strain on cytokine responses and scar frequency.

Cytokine	Assay stimulus	Russia (*n* = 719)	Bulgaria (*n* = 508)		Denmark (*n* = 114)	
		Geometric mean (pcg/ml)	Geometric mean (pcg/ml)	GMR^a^ (95% CI)	Geometric mean (pcg/ml)	GMR^a^ (95% CI)
IFN-γ	cCFP^b^	261.9	262.8	0.96 (0.61, 2.04)	622.3	**2.24 (1.42, 4.00)**
	Ag85	130.9	113.3	0.80 (0.46, 1.32)	278.3	**2.01 (1.38, 2.69)**
	TT^c^	28.5	31.9	0.96 (0.46, 2.04)	82.6	**2.41 (1.14, 4.85)**
	PHA	281.2	299.3	1.00 (0.62, 1.66)	891.4	**3.09 (2.11, 5.71)**

IL-5	cCFP^b^	4.4	3.3	**0.70 (0.23, 0.98)**	5.2	1.13 (0.81, 1.66)
	Ag85	2.5	2.2	**0.83 (0.71, 0.99)**	3.0	1.15 (0.48, 1.82)
	TT^c^	11.1	9.1	0.61 (0.23, 1.34)	16.3	0.92 (0.34, 2.19)
	PHA	43.3	28.5	0.61 (0.35, 1.04)	93.1	2.01 (0.98, 3.81)

IL-13	cCFP^b^	19.4	14.4	0.74 (0.35, 1.32)	30.7	**1.51 (1.14, 2.43)**
	Ag85	7.7	6.5	0.81 (0.52, 1.08)	10.4	1.27 (0.54, 2.02)
	TT^c^	43.1	32.0	0.69 (0.31, 1.51)	51.3	0.95 (0.38, 2.02)
	PHA	140.4	137.5	0.96 (0.60, 1.81)	342.5	**2.28 (1.55, 4.14)**

IL-10	cCFP^b^	56.3	119.7	**2.17 (1.48, 4.03)**	234.3	**4.16 (2.85, 6.89)**
	Ag85	49.1	103.4	**2.06 (1.27, 2.82)**	78.3	**1.52 (1.10, 2.21)**
	TT^c^	3.3	8.6	**2.17 (1.33, 8.67)**	10.3	**2.59 (1.39, 6.08)**
	PHA	125.7	203.8	1.53 (0.99, 2.38)	360.3	**2.79 (2.10, 4.24)**

Russia (*n* = 719)	Bulgaria (*n* = 508)		Denmark (*n* = 114)	
BCG scar frequency (%)	BCG scar frequency (%)	Scar presence OR (95% CI)	BCG scar frequency (%)	Scar presence OR (95% CI)
352 (49.0%)	333 (65.6%)	**1.84 (1.25, 2.71)**	106 (92.3%)	**14.28 (5.85, 34.85)**

The table shows strain-specific mean cytokine responses (IFN-γ, IL-5, IL-13 and IL-10) to four assay stimuli: culture filtrate protein (cCFP); antigen 85 (Ag85); tetanus toxoid (TT); and phytohaemagglutinin (PHA). Scar frequencies and proportions (%) for each BCG-strain group are also included as well as odds ratios (ORs) for scar presence. Missing values for cCFP^b^, Ag85, TT and PHA assays are 252, 1, 7 and 15 respectively.

a Geometric mean ratios (GMRs) comparing responses in infants vaccinated with BCG-Bulgaria or BCG-Denmark versus BCG-Russia, with bias-corrected accelerated confidence intervals. These are adjusted for: study area, infant malaria parasitaemia, infant HIV status, maternal *M. perstans* infection and maternal hookworm infection (at 1 year). A random effects model was applied to the regression calculations to allow for clustering within BCG lot groups. GMRs and ORs significantly different from 1.00 are indicated in bold.

b Excluding the 2nd batch of cfp antigen (see text).

c Adjusted for tetanus toxoid batch (see text).

**Table 3:** Sex-stratified effect of BCG strain on cytokine responses to tetanus toxoid (TT) and BCG scar frequency.

Cytokine	Sex (M/F)	Russia (*n* = 719)	Bulgaria (*n* = 508)		Denmark (*n* = 114)		Interaction *p*-value^b^
		Geometric mean (pcg/ml)	Geometric mean (pcg/ml)	GMR^a^ (95% CI)	Geometric mean (pcg/ml)	GMR^a^ (95% CI)	
IFN-γ	M	34.2	1.04	0.97 (0.53, 1.77)	59.2	1.31 (0.47, 3.63)	0.083
	F	23.9	25.23	0.88 (0.47, 1.66)	115.3	**4.14 (1.47, 11.61)**	

IL-5	M	12.3	11.63	0.64 (0.37, 1.13)	13.6	0.60 (0.23, 1.54)	0.201
	F	10.0	6.94	0.57 (0.32, 1.01)	19.7	1.35 (0.52, 3.48)	

IL-13	M	46.1	38.29	0.69 (0.42, 1.16)	35.1	0.50 (0.21, 1.19)	0.056
	F	40.4	26.28	0.67 (0.39, 1.13)	74.9	1.74 (0.73, 4.13)	

IL-10	M	4.1	9.59	**1.74 (1.09, 2.78)**	6.7	1.08 (0.49, 2.37)	0.01
	F	2.7	7.70	**2.73 (1.71, 4.36)**	15.9	**6.73 (3.13, 14.45)**	

Sex (M/F)	Russia (*n* = 719)	Bulgaria (*n* = 508)		Denmark (*n* = 114)		*p*-Value^c^
	BCG scar frequency (%)	BCG scar frequency (%)	Scar presence OR (95% CI)	BCG scar frequency (%)	Scar presence OR (95% CI)	
M	182 (51.9%)	173 (65.3%)	**1.64 (1.04, 2.60)**	53 (93.0%)	**12.40 (3.85, 39.96)**	0.557
F	170 (46.3%)	160 (65.8%)	**2.18 (1.36, 3.49)**	53 (93.0%)	**16.43 (5.12, 52.74)**	

The table shows strain-specific cytokine responses (IFN-γ, IL-5, IL-13 and IL-10) to tetanus toxoid (TT) antigen and BCG scar frequency in each BCG strain group, stratified by sex. There are 6 missing values (3 males and 3 females). Sex did not significantly interact with cytokine responses to cCFP, Ag85 or PHA (data not shown).

a Geometric mean ratios (GMRs) comparing responses in infants vaccinated with BCG-Bulgaria or BCG-Denmark versus BCG-Russia, with bias-corrected accelerated confidence intervals. These are adjusted for: study area, malaria parasitaemia, HIV status, *M. perstans* infection and hookworm infection (at 1 year). A random effects model was applied to the regression calculations to allow for clustering within BCG lot groups. GMRs and ORs significantly different from 1.00 and *p*-values that are <0.05 are indicated in bold.

b *p*-Value for test of null hypothesis that there is no interaction of sex with the effect of strain on cytokine responses to tetanus toxoid.

c *p*-Value for test of null hypothesis that there is no interaction of sex with the effect of strain on BCG scar frequency.

**Table 4:** Relationship between cytokine responses and BCG scar presence.

Cytokine	Assay stimulus	Absent scar (*n* = 549)	Present scar (*n* = 791)	
		Geometric mean (pcg/ml)	Geometric mean (pcg/ml)	GMR^a^ (95% CI)
IFN-γ	cCFP^b^	214.4	339.9	**1.59 (1.21, 2.10)**
	Ag85	92.0	170.1	**1.85 (1.43, 2.42)**
	TT^c^	27.9	36.3	1.24 (0.92, 1.68)
	PHA	302.1	328.1	1.09 (0.87, 1.36)

IL-5	cCFP^b^	3.8	4.4	1.17 (0.91, 1.50)
	Ag85	2.2	2.5	1.15 (0.95, 1.39)
	TT^c^	37.2	41.0	1.10 (0.83, 1.47)
	PHA	9.8	11.2	1.11 (0.84, 1.48)

IL-13	cCFP^b^	14.0	22.8	**1.63 (1.26, 2.10)**
	Ag85	5.9	8.7	**1.46 (1.17, 1.82)**
	TT^c^	39.2	38.9	0.97 (0.76, 1.26)
	PHA	141.6	156.2	1.10 (0.91, 1.36)

IL-10	cCFP^b^	75.3	77.2	1.02 (0.82, 1.29)
	Ag85	64.2	70.4	1.10 (0.89, 1.35)
	TT^c^	5.0	5.5	1.00 (0.79, 1.26)
	PHA	160.5	168.0	1.05 (0.87, 1.26)

The table compares the cytokine responses of infants, with and without BCG scars, to four assay stimuli: crude culture filtrate protein (cCFP); antigen 85 (Ag85); tetanus toxoid (TT); and phytohaemagglutinin (PHA). Missing values for cCFP^b^, Ag85, TT and PHA assays are 253, 2, 8 and 16 respectively.

a Geometric mean ratios (GMRs) comparing those with scars to those without are shown with bias-corrected accelerated confidence intervals. GMRs significantly different from 1.00 are indicated in bold.

b Excluding the 2nd batch of cCFP antigen (see text).

c Adjusted for TT batch (see text).

**Table 5:** Effect of BCG strain on mortality.

BCG strain group	Number of deaths from all causes	Rate per 1000 person years (95% CI)	Hazard ratio (95% CI)	*p*-Value
Russia (*n* = 1124)	22	20.5 (13.5, 31.1)	1	0.87
Bulgaria (*n* = 788)	14	18.6 (11.0, 31.4)	0.91 (0.46, 1.77)	
Denmark (*n* = 169)	4	25.0 (9.4, 66.7)	1.22 (0.42, 3.53)	

## Footnotes

2 See supplementary information for BCG lot numbers used.

3 The cut-off points for a positive cytokine response were set to IFN-γ = 73 pg/ml; IL-5 = 34 pg/ml; IL-13 = 8 pg/ml; IL-10 = 48 pg/ml (the mean level plus two standard deviations of the negative control).
