# Supplementary material for: The influence of BCG vaccine strain on mycobacteria-specific and non-specific immune responses in a prospective cohort of infants in Uganda
Source: Vaccine. 2012 Mar 9;30(12):2083–9. doi: 10.1016/j.vaccine.2012.01.053 (PMC3314967; doi:10.1016/j.vaccine.2012.01.053)
Supplement: Supplementary file 1 [file mmc1.doc]

**Supplementary information**

| BCG lot numbers^a^ | | |
| --- | --- | --- |
| BCG-Russia | BCG-Bulgaria | BCG-Denmark |
| EU20221 | 841 | 104048A |
| EU20224 | 841 | 104071A |
| EU20228 | 939 | 105090A |
| EU20229 | 974-2 | 106018A |
| EU20230 | 113-1 |  |
| EU20239 | 139-2 |  |
| EU20280 | 162-1 |  |
| EU20303 | 163-1 |  |
| EU20304 | 162-2 |  |
|  | 163-1 |  |
|  | 162-1 |  |
| ^a^Infants were immunised at birth with the BCG strain and lot available at the time. A random effects model was used to account for potential between-lot variability in analyses comparing strains | | |
